# Dosimetric comparison of TomoTherapy and non-coplanar VMAT for hippocampal-avoidance prophylactic cranial irradiation

**DOI:** 10.3389/fonc.2026.1790929

**Published:** 2026-03-23

**Authors:** Young Kyu Lee, Wonjoong Cheon, Yunji Seol, Chan-beom Park, Geum Seong Cheon, Jin-Ho Song, Ji Hyun Hong, Young Nam Kang, Yeon-Sil Kim, Kyu Hye Choi

**Affiliations:** 1 Department of Radiation Oncology, Seoul St. Mary’s Hospital, College of Medicine, The Catholic University of Korea, Seoul, Republic of Korea; 2 Department of Biomedicine and Health Sciences, College of Medicine, The Catholic University of Korea, Seoul, Republic of Korea

**Keywords:** cognitive function preservation, dosimetric comparison, hippocampal avoidance, non-coplanar technique, prophylactic cranial irradiation, small cell lung cancer, TomoTherapy, volumetric modulated arc therapy

## Abstract

**Purpose:**

To compare the dosimetric characteristics of non-coplanar volumetric modulated arc therapy (VMAT) and TomoTherapy for hippocampal-avoidance prophylactic cranial irradiation (HA-PCI) in small cell lung cancer (SCLC) patients.

**Methods:**

Ten SCLC patients who received HA-PCI were retrospectively analyzed. Two plans were generated as TrueBeam non-coplanar VMAT with four arcs and helical TomoTherapy. The hippocampal avoidance zone used a 3-mm margin, reduced from RTOG 0933’s 5-mm specification. Planning target volume of whole brain (PTV_WB) was prescribed 25 Gy in 10 fractions, normalized to D_95%_=2500 cGy. RTOG 0933 hippocampal constraints (D_max_ ≤ 1600 cGy, D_100%_≤900 cGy) were applied. Dosimetric parameters for hippocampus, PTV_WB, organs at risk, treatment efficiency (monitor units, delivery time), Paddick conformity index, and homogeneity index were compared using Wilcoxon signed-rank test.

**Results:**

Non-coplanar VMAT achieved significantly lower hippocampal D_max_ than TomoTherapy (1353 cGy *vs* 1638 cGy, p=0.004), meeting RTOG 0933 constraints, while TomoTherapy exceeded the per-protocol constraint by 38 cGy but remained within the acceptable deviation threshold, indicating clinically acceptable dosimetric outcomes. Non-coplanar VMAT demonstrated superior PTV_WB coverage: V_98%_ (96.68% *vs* 95.77%), V_95%_ (97.66% *vs* 96.48%), D_98%_ (2320 cGy *vs* 2095 cGy) (all p=0.004). Paddick conformity index was higher (0.91 *vs* 0.84, p=0.012) and homogeneity index lower (0.20 *vs* 0.27, p=0.004). Non-coplanar VMAT reduced monitor units by 88.5% (748 *vs* 6528 MU, p=0.004) and treatment time by 25.2% (287 *vs* 384 seconds, p=0.004). Bilateral eye D_max_ was 21-27% lower (all p=0.004) and bilateral cochlear D_max_ approximately 15% lower (p ≤ 0.008).

**Conclusions:**

Non-coplanar VMAT demonstrated superior dosimetric characteristics compared to TomoTherapy for HA-PCI with 3-mm margin, meeting hippocampal constraints while improving target coverage and treatment efficiency. Prospective validation with neurocognitive outcomes is needed.

## 1 Introduction

Small cell lung cancer (SCLC) accounts for approximately 13-15% of all lung cancers (1, 2). At diagnosis, 10-20% of patients present with brain metastases, and up to 50-60% develop brain metastases during treatment (3, 4). Prophylactic cranial irradiation (PCI) is a standard treatment that reduces brain metastasis incidence (relative risk of 0.46 for brain metastasis reduction) and improves overall survival, delivered as 25 Gy in 10 fractions (5, 6).

However, whole-brain radiotherapy causes neurocognitive dysfunction including memory impairment and learning deficits (7–9). The hippocampus is critical for memory formation and learning, containing radiation-sensitive neural stem cells in the subgranular zone. Radiation exposure suppresses neurogenesis and damages cognitive function through direct neural stem cell injury and microenvironmental disruption (10–12). Hippocampal-avoidance whole-brain radiotherapy (HA-WBRT) was developed to address this, with cognitive preservation benefits demonstrated in RTOG 0933 and NRG CC001 trials (13, 14).

RTOG 0933 established a hippocampal avoidance zone using a 5-mm margin with dose constraints of D_max_ ≤ 16 Gy and D_100%_≤9 Gy (15). Although PCI prescription dose (25 Gy in 10 fractions) is lower than RTOG 0933 (30 Gy in 10 fractions), we applied identical hippocampal constraints based on the biological principle that hippocampal neural stem cell damage depends on absolute dose rather than fractionation schedule (11, 12).

Recent advances in IGRT with daily cone-beam CT and submillimeter positioning accuracy have enabled margin reduction. High-precision OAR contouring following consensus guidelines is essential for achieving tight margins while maintaining adequate hippocampal protection (15–17).

A 3-mm hippocampal margin minimizes the hippocampal avoidance zone, increasing the whole-brain planning target volume (PTV_WB). This margin reduction aims to enhance prophylactic coverage of potential microscopic disease while maintaining adequate hippocampal protection. However, margin reduction demands substantially steeper dose gradients between the hippocampus and PTV_WB, presenting a challenge to the geometric capabilities of treatment delivery techniques.

TomoTherapy (Accuray Inc., Sunnyvale, CA, USA) is a helical delivery system that employs continuous 360-degree gantry rotation with simultaneous couch translation, enabling highly conformal dose distributions through binary multileaf collimator (MLC) modulation (18–20).

Various treatment techniques have been compared for achieving hippocampal protection in hippocampal-avoidance whole-brain radiotherapy, and multiple studies have demonstrated that TomoTherapy provides superior hippocampal sparing compared to other techniques. Gondi et al. reported in the RTOG 0933 pilot study that TomoTherapy achieved lower hippocampal doses compared to LINAC-based IMRT (15). Rong et al. concluded in a dosimetric comparison study that TomoTherapy provided superior homogeneity index compared to IMRT and VMAT and was the preferred modality for hippocampal-avoidance whole-brain radiotherapy (21). Similarly, Zhang et al. reported in a recent study comparing helical tomotherapy and VMAT that TomoTherapy possessed dosimetric advantages in multi-angle beam delivery and strong intensity modulation capability (22).

Specifically in hippocampal-avoidance prophylactic cranial irradiation for small cell lung cancer patients, Yetmen Dogan et al. also compared TomoTherapy and coplanar VMAT. In their treatment planning study using identical prescription (25 Gy in 10 fractions) and standard 5-mm hippocampal margins, TomoTherapy achieved significantly lower hippocampal maximum dose compared to coplanar VMAT (D_max_: 15.91 Gy *vs* 20.47 Gy, p<0.001). Importantly, even with 5-mm margins, TomoTherapy approached very close to the RTOG 0933 constraint (D_max_ ≤ 16 Gy), reaching 15.91 Gy (99.4% of the constraint). Coplanar VMAT exceeded the constraint by 4.47 Gy (28%), demonstrating inadequacy for hippocampal protection (23).

These studies consistently demonstrate that TomoTherapy provides superior hippocampal protection compared to coplanar IMRT and VMAT techniques in hippocampal-avoidance whole-brain radiotherapy using standard 5-mm margins.

However, these findings have important implications for the impact of margin reduction on dosimetric performance. Although TomoTherapy achieved a hippocampal maximum dose of 15.91 Gy while maintaining adequate target coverage with 5-mm margins, this represents 99.4% of the RTOG 0933 constraint (D_max_ ≤ 16 Gy), leaving only very limited margin (23). A 40% margin reduction from 5 mm to 3 mm substantially reduces the distance between the hippocampus and PTV_WB, demanding steeper dose gradients. Under these geometric constraints, the 3-mm margin is likely to drive the hippocampal maximum dose to exceed the 16 Gy constraint.

This presents an important dosimetric challenge in HA-PCI using reduced margins. When the hippocampal maximum dose exceeds the constraint, TomoTherapy may face difficulty in simultaneously achieving adequate target coverage (D_95%_ = 2500 cGy) and hippocampal constraints (D_max_ ≤ 1600 cGy). In this situation, meeting the hippocampal constraint may require reducing target coverage below optimal levels, creating a clinical trade-off between prophylactic efficacy and cognitive preservation. Therefore, systematic evaluation of alternative treatment delivery approaches capable of overcoming this dosimetric challenge is needed.

Coplanar VMAT already substantially exceeded the constraint with 5-mm margins (20.47 Gy) (23), so even more inferior hippocampal protection is expected with 3-mm margins. Importantly, this limitation arises not from the number of arcs or optimization parameters, but from fundamental constraints of beam geometry. While both TomoTherapy and coplanar VMAT predominantly deliver beams from the axial or transverse direction, the longitudinally-oriented hippocampal structures require dose sculpting in the superior-inferior direction. This geometric mismatch creates constraints in achieving steep dose gradients with reduced margins.

These limitations necessitate evaluation of alternative delivery approaches that transcend axial beam geometry. TrueBeam (Varian Medical Systems, Palo Alto, CA, USA) non-coplanar VMAT increases beam entrance pathway diversity by employing multiple couch angles (typically 0°, 315°, 45°, and 90°) (24, 25). This geometric flexibility enables superior three-dimensional dose sculpting capability, allowing beams to avoid the hippocampus from multiple geometric perspectives while maintaining adequate coverage of the surrounding PTV_WB.

Specifically, couch angles other than 0° enable dose sculpting in the superior-inferior direction. A 90°couch angle provides tangential beam geometry for longitudinally-oriented hippocampal structures, potentially enhancing the ability to achieve the steep dose gradients required with reduced margins. This enhanced geometric capability may potentially overcome the target coverage versus hippocampal protection trade-off anticipated with TomoTherapy’s predominantly axial beam arrangement.

Previous studies primarily focused on brain metastasis patients using 5-mm margins or evaluated single systems without direct head-to-head comparisons (21, 26). No study has systematically compared TomoTherapy and non-coplanar VMAT in PCI patients using 3-mm margins. Moreover, the ability of each technique to meet hippocampal constraints while maintaining sufficient target coverage under reduced margins—specifically without target under-dosing—has not yet been characterized.

Therefore, this study provides the first direct comparison of TomoTherapy and TrueBeam non-coplanar VMAT for HA-PCI with 3-mm hippocampal margins in SCLC patients. The primary objective of this study is to evaluate whether non-coplanar VMAT can meet hippocampal constraints while maintaining adequate target coverage, and whether it can overcome the target coverage versus hippocampal protection trade-off compared to TomoTherapy.

Specifically, we evaluated the following under identical target coverage conditions (D_95%_ = 2500 cGy): (i) hippocampal protection and RTOG 0933 constraint compliance, (ii) PTV_WB coverage and dose homogeneity, (iii) organ-at-risk doses, (iv) treatment efficiency.

We hypothesized that non-coplanar VMAT’s superior three-dimensional geometric flexibility would enable better hippocampal sparing with reduced margins compared to TomoTherapy’s predominantly axial beam arrangement, achieving hippocampal constraint compliance without sacrificing target coverage. This evidence will inform optimal treatment technique selection for high-precision HA-PCI with reduced margins.

## 2 Materials and methods

### 2.1 Study design and ethical approval

This single-institution retrospective treatment planning comparison study compared the dosimetric characteristics of TomoTherapy and TrueBeam non-coplanar VMAT for hippocampal-avoidance PCI in SCLC patients. The paired comparison design, where each patient served as their own control with two different planning techniques applied to identical anatomy, minimized inter-patient variability and enhanced statistical power. The study was approved by the Institutional Review Board (approval number: KC24RISI0078).

### 2.2 Patient selection and image acquisition

Ten patients diagnosed with SCLC who received HA-PCI with TomoTherapy following primary treatment response between October 2023 and December 2024 were included. All patients met the indication criteria for prophylactic cranial irradiation. Sample size of 10 patients was determined based on institutional feasibility and is consistent with previous dosimetric comparison studies in hippocampal-avoidance radiotherapy. The paired study design, where each patient serves as their own control, enhances statistical power and minimizes inter-patient anatomical variability.

All patients were immobilized using individually customized thermoplastic masks. Non-contrast CT images with 1-mm slice thickness were acquired using a SOMATOM go.Open Pro CT scanner (Siemens Healthineers, Germany). Additional T1-weighted non-contrast MRI with 1-mm isotropic resolution was acquired for hippocampal contouring and rigidly registered to CT. Patient demographic and clinical characteristics are summarized in **Table 1**.

**Table 1:** Patient demographic and clinical characteristics.

Characteristic	Value (N = 10)
Age, median (range), years	65 (59-76)
Sex, n (%)	
Male	9
Female	1
ECOG performance status, n (%)	
0-1	10
2	0
Smoking history, n (%)	
Current	5
Former	5
Never	0
Disease stage, n (%)	
Limited	9
Extensive	1
Initial treatment, n (%)	
Chemo-RT	8
Chemo alone	2
Response to initial treatment, n (%)	
Complete response	8
Partial response	1
Stable disease	1
Time from primary treatment to HA-PCI, median (range), months	4.8 (3.2-8.0)

ECOG, Eastern Cooperative Oncology Group; Chemo-RT, Concurrent Chemoradiotherapy; HA-PCI, Hippocampal-Avoidance Prophylactic Cranial Irradiation.

### 2.3 Target volume and organ-at-risk delineation

hippocampal contours were delineated on T1-weighted non-contrast MRI following RTOG 0933 guidelines (15). The hippocampal avoidance zone was created by isotropically expanding the hippocampal contour by 3 mm. PTV_WB was defined as the entire brain minus the hippocampal avoidance zone. Organs at risk included bilateral hippocampi, lenses, eyes, optic nerves, optic chiasm, cochleae, and parotid glands.

### 2.4 Radiation treatment planning

All treatment plans prescribed 25 Gy in 10 fractions to PTV_WB, normalized so that 95% of PTV_WB received 100% of the prescription dose (D_95%_=2500 cGy). Both planning techniques prioritized PTV_WB coverage while respecting RTOG 0933 hippocampal dose constraints (D_max_ ≤ 16 Gy, D_100%_≤9 Gy). Since the hippocampus contains abundant radiation-sensitive neural stem cells whose depletion correlates with cognitive decline independent of fractionation (11, 12), we applied absolute dose constraints from RTOG 0933 to PCI despite the lower prescription dose.

This study utilized actual clinical patient CT images and organ contours from patients who underwent HA-PCI treatment. For each patient, TomoTherapy treatment plans represented clinically delivered treatments, while non-coplanar VMAT plans were generated retrospectively for comparison using identical CT datasets, target volumes, and organ-at-risk delineations. This paired comparison approach enabled direct dosimetric evaluation of the two techniques while eliminating inter-patient anatomical variability as a confounding factor.

#### 2.4.1 TomoTherapy planning

Precision treatment planning system version 1.1.1.1 (Accuray Inc., USA) was used. Helical tomotherapy employed 6-MV photon beams with a 64-leaf binary MLC. Field width was 2.5 cm, pitch 0.43, and modulation factor was individually optimized for each patient (range: 1.8-2.4). Dose calculation used the convolution-superposition algorithm at medium resolution with tissue heterogeneity corrections.

TomoTherapy plans were based on clinically delivered treatment plans from our institutional practice. Planning parameters were established through clinical optimization during initial implementation of hippocampal-avoidance WBRT at our institution, balancing dosimetric quality with treatment efficiency. All plans were evaluated against RTOG 0933 protocol criteria, which define both per-protocol constraints (hippocampal D_max_ ≤ 16 Gy) and acceptable deviation thresholds (hippocampal D_max_ ≤ 17 Gy).

#### 2.4.2 TrueBeam non-coplanar VMAT planning

TrueBeam non-coplanar VMAT treatment planning was performed using Eclipse treatment planning system version 16.0 (Varian Medical Systems, Palo Alto, USA). Planning was based on a TrueBeam linear accelerator equipped with a Millennium 120 multi-leaf collimator (MLC), using 6-MV photon beams.

The non-coplanar technique was implemented based on the HyperArc template, utilizing a total of 4 arcs. Each arc’s gantry was set to rotate as follows: 180.1°-179.9° (clockwise), 179.9°-0° (counterclockwise), 0°-180.1° (counterclockwise), and 180.1°-0° (clockwise). Non-coplanar beam arrangements were achieved by rotating the treatment couch to angles of 0°, 315°, 45°, and 90°, respectively. These couch angles represent the standard configuration of the HyperArc template, which provides three-dimensional beam trajectory diversity: the 0° angle delivers standard coplanar beams as a baseline, oblique angles (315° and 45°) enable tangential avoidance of the longitudinally-oriented hippocampal structures, and the 90° angle facilitates superior-inferior dose sculpting along the hippocampal axis — a geometric advantage particularly critical for achieving steep dose gradients with the reduced 3-mm margin. Collimator angles were optimized according to each patient’s anatomical structure.

Treatment plan optimization was performed using the Photon Optimizer (PO) algorithm. Control points were generated at 2-degree angular spacing, resulting in 180 control points for the full-rotation arc (360°) and 90 control points for each partial-rotation arc (180°), totaling 450 control points across all 4 arcs. This control point density provides high-resolution dose modulation comparable to TomoTherapy’s binary MLC system. Final dose calculation was performed using the Anisotropic Analytical Algorithm (AAA) with a dose calculation grid size of 1.25 mm. All plans were evaluated against the same RTOG 0933 protocol criteria used for TomoTherapy assessment.

#### 2.4.3 Quality assurance

Target volumes and organs-at-risk were delineated by board-certified radiation oncologists and independently peer-reviewed by a second radiation oncologist to ensure consistency with institutional protocols.

Treatment planning was performed by medical physicists with more than 10 years of clinical treatment planning experience. All plans underwent institutional quality assurance procedures, including independent review by medical physicists and radiation oncologists, to verify dosimetric quality and protocol compliance.

### 2.5 Evaluation methods

#### 2.5.1 Target volume evaluation

PTV_WB dose coverage was evaluated using V_98%_, V_95%_, D_98%_, D_50%_, and D_2%_. Paddick conformity index (CI) was calculated as (Equation 1):

(1) $$\text{CI}=\frac{TV_{RI}^{2}}{TV\times V_{RI}}\;$$

where TV_RI_ is the target volume covered by the prescription isodose line, TV is the target volume (PTV_WB), and V_RI_ is the total volume enclosed by the prescription isodose line. This formula penalizes both target under coverage and excessive normal tissue irradiation, with values closer to 1 indicating superior conformity.

Homogeneity index (HI) was calculated as (Equation 2):

(2) $$\text{HI}=\frac{D_{2\%}-D_{98\%}}{D_{50\%}}$$

where D_2%_ represents near-maximum dose received by 2% of PTV_WB, D_98%_ represents near-minimum dose received by 98% of PTV_WB, and D_50%_ represents median dose. Lower HI values indicate more uniform dose distribution within PTV_WB.

#### 2.5.2 Organ-at-risk dose and treatment efficiency evaluation

Bilateral hippocampal D_max_, D_mean_, and D_100%_ were analyzed to verify compliance with RTOG 0933 constraints (D_max_ ≤ 16 Gy, D_100%_≤9 Gy). Additional organs at risk were evaluated: maximum doses to bilateral optic nerves, eyes, and lenses, and maximum doses to bilateral cochleae and parotid glands.

Treatment efficiency was assessed using total monitor units (MU) and treatment delivery time. For TomoTherapy, beam-on time calculated by the treatment planning system was recorded. For TrueBeam non-coplanar VMAT, total treatment time from first beam-on to final beam-off was recorded, including all mechanical movements.

### 2.6 Statistical analysis

All dosimetric parameters are presented as mean ± standard deviation. Comparisons between TomoTherapy and non-coplanar VMAT used the Wilcoxon signed-rank test. Given the small sample size (n=10), this nonparametric test was selected without assumption of normal distribution, which is appropriate for paired comparisons where each patient serves as their own control. Statistical significance was defined as p<0.05 in two-tailed tests. A *post-hoc* power analysis was performed for the primary endpoint of hippocampal D_max_ using a paired t-test framework. Based on the observed mean difference of 285.32 cGy and standard deviation of differences of 114.68 cGy (Cohen’s d = 2.488), the achieved statistical power was 100% at a significance level of α = 0.05 (two-tailed), confirming that the sample size of n = 10 was sufficient to detect the observed differences with high statistical confidence. Statistical analyses were performed using SPSS version 26.0 (IBM Corp., USA) and Python 3.9 SciPy package.

## 3 Results

### 3.1 Target volume evaluation

**Figure 1:**
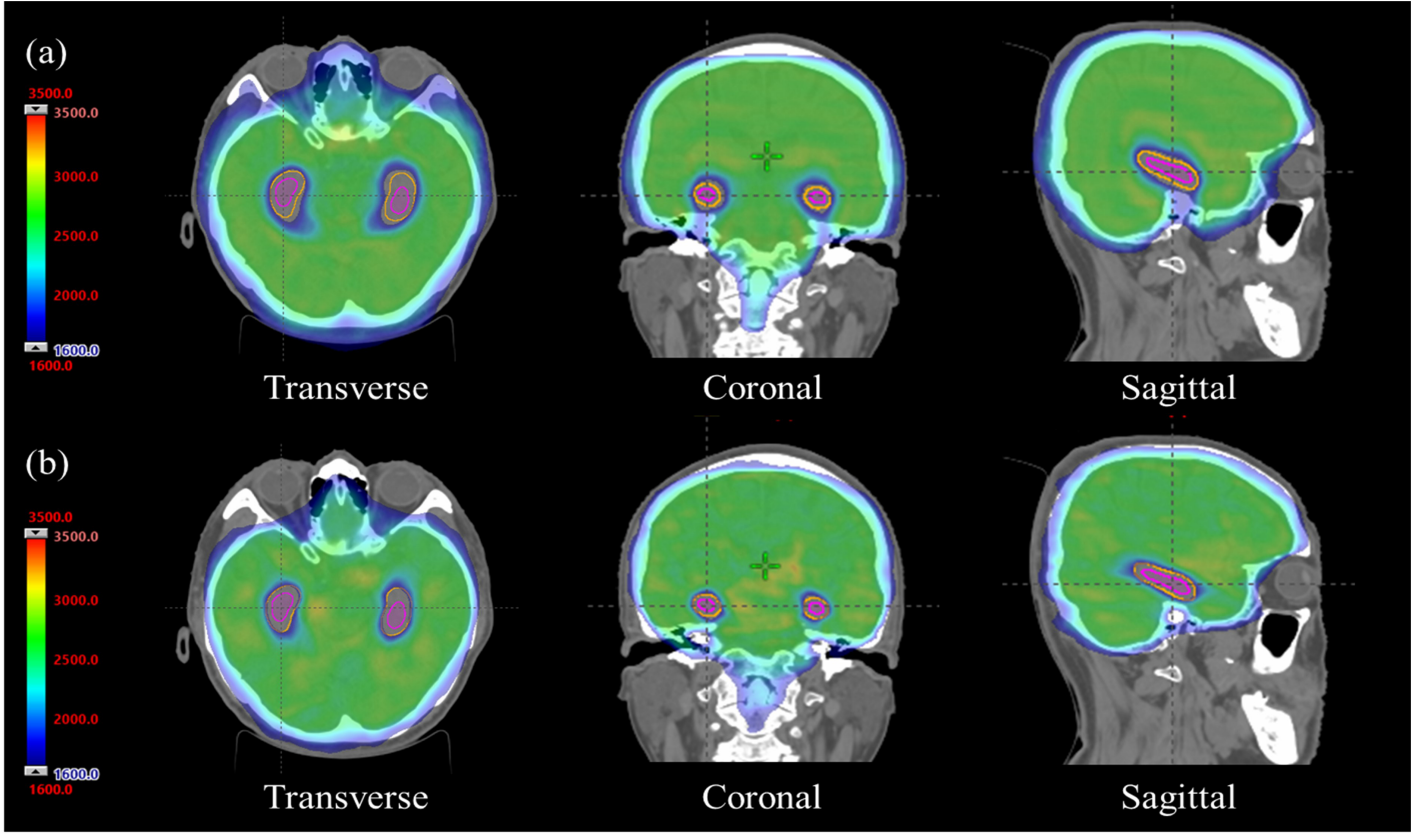
Representative dose distributions comparing **(a)** TomoTherapy and **(b)** non-coplanar volumetric modulated arc therapy (VMAT) in transverse, coronal, and sagittal views. Purple contours indicate hippocampi; orange contours show 3-mm hippocampal avoidance zones. The green region represents the prescription isodose (2500 cGy). Note superior hippocampal sparing with non-coplanar VMAT while maintaining excellent PTV_WB coverage. Both plans were normalized to D_95%_=2500 cGy.

**Figure 1** shows representative dose distributions. **Table 2** presents dosimetric parameters for PTV_WB. Non-coplanar VMAT was statistically significantly superior to TomoTherapy in prescription dose coverage: V_98%_ was 96.68 ± 0.29% versus 95.77 ± 0.19% (p=0.004), V_95%_ was 97.66 ± 0.34% versus 96.48 ± 0.30% (p=0.004), and D_98%_ was 2319.87 ± 58.82 cGy versus 2095.27 ± 65.12 cGy (p=0.004).

**Table 2:** Dosimetric comparison of PTV_WB between TomoTherapy and non-coplanar VMAT.

Parameter	TomoTherapy	Non-coplanar VMAT	p-value
V_98%_(%)	95.77 ± 0.19	96.68 ± 0.29	0.004
V_95%_(%)	96.48 ± 0.30	97.66 ± 0.34	0.004
D_98%_ (cGy)	2095.27 ± 65.12	2319.87 ± 58.82	0.004
D_50%_ (cGy)	2710.72 ± 78.53	2645.91 ± 26.14	0.008
D_2%_ (cGy)	2833.29 ± 88.29	2845.72 ± 69.46	0.652
CI	0.84 ± 0.06	0.91 ± 0.01	0.012
HI	0.27 ± 0.05	0.20 ± 0.04	0.004

CI, conformity index; HI, homogeneity index.

Target median dose D_50%_ was significantly higher in TomoTherapy at 2710.72 ± 78.53 cGy compared to 2645.91 ± 26.14 cGy in non-coplanar VMAT (p=0.008). Near-maximum dose D_2%_ showed no significant difference between techniques (2833.29 ± 88.29 cGy versus 2845.72 ± 69.46 cGy, p=0.652).

Paddick conformity index was significantly higher in non-coplanar VMAT at 0.91 ± 0.01 compared to 0.84 ± 0.06 in TomoTherapy (p=0.012). Homogeneity index was significantly lower in non-coplanar VMAT at 0.20 ± 0.04 compared to 0.27 ± 0.05 in TomoTherapy (p=0.004), demonstrating more uniform dose distribution within PTV_WB.

### 3.2 Organ-at-risk dose evaluation

**Figure 2:**
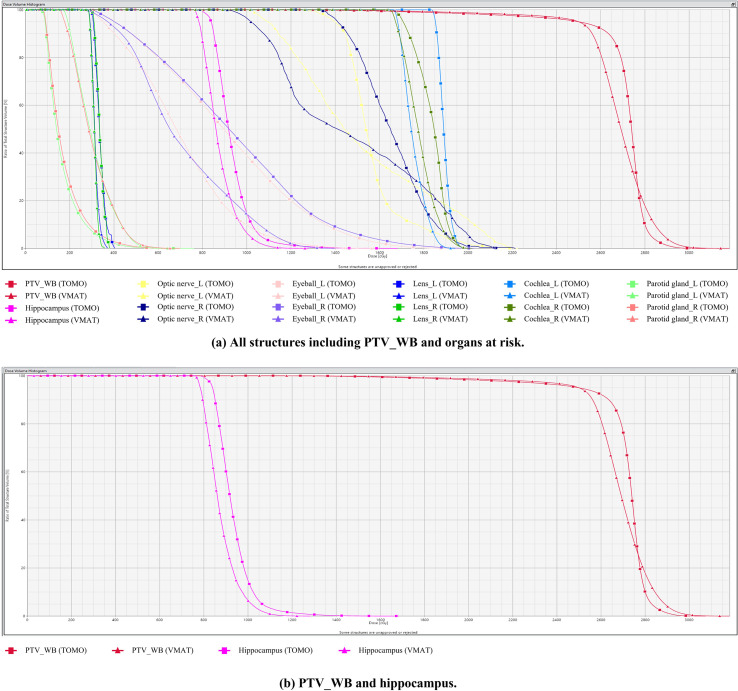
Dose-volume histograms comparing TomoTherapy (squares) and non-coplanar volumetric modulated arc therapy (VMAT) (triangles). **(a)** All structures including PTV_WB and organs at risk. **(b)** PTV_WB and hippocampus.

**Table 3** presents hippocampal and organ-at-risk doses. **Figure 2** shows representative dose-volume histograms for all structures (a) and for PTV_WB and hippocampus (b).

**Table 3:** Dose comparison for hippocampus and organs at risk between TomoTherapy and non-coplanar VMAT.

Structure	Parameter	TomoTherapy	Non-coplanar VMAT	p-value
Hippocampus	D_max_ (cGy)	1638.03 ± 96.22	1352.71 ± 78.07	0.004
	D_mean_ (cGy)	1043.33 ± 104.42	935.39 ± 49.08	0.004
	D_100%_ (cGy)	899.80 ± 108.56	763.33 ± 31.45	0.004
Optic nerve	Left D_max_ (cGy)	2404.50 ± 433.44	2113.70 ± 218.75	0.045
	Right D_max_ (cGy)	2398.37 ± 481.59	2071.40 ± 253.06	0.043
Eyeball	Left D_max_ (cGy)	1981.09 ± 92.80	1556.83 ± 138.72	0.004
	Right D_max_ (cGy)	2116.07 ± 119.94	1547.29 ± 175.95	0.004
Lens	Left D_max_ (cGy)	453.08 ± 101.04	350.33 ± 69.26	0.041
	Right D_max_ (cGy)	461.58 ± 106.43	367.97 ± 73.00	0.04
Cochlea	Left D_max_ (cGy)	2054.39 ± 514.79	1739.61 ± 364.00	0.004
	Right D_max_ (cGy)	2051.19 ± 469.51	1728.54 ± 358.04	0.008
Parotid gland	Left D_mean_ (cGy)	218.18 ± 148.67	325.21 ± 94.29	0.034
	Right D_mean_ (cGy)	217.67 ± 133.74	314.48 ± 80.89	0.027

#### 3.2.1 Hippocampal protection

For hippocampal sparing, TomoTherapy plans exceeded the RTOG 0933 per-protocol constraint of 16 Gy (mean D_max_: 1638.03 ± 96.22 cGy) but remained within the acceptable deviation threshold of 17 Gy, indicating clinically acceptable dosimetric outcomes. In contrast, non-coplanar VMAT plans achieved per-protocol compliance with substantial margin (mean D_max_: 1352.71 ± 78.07 cGy), demonstrating statistically significant superior hippocampal sparing with 285.32 cGy absolute dose reduction (17.4% relative reduction, p=0.004).

Non-coplanar VMAT demonstrated statistically significant superiority over TomoTherapy in all hippocampal dose parameters. Mean hippocampal dose (D_mean_) was 935.39 ± 49.08 cGy for non-coplanar VMAT versus 1043.33 ± 104.42 cGy for TomoTherapy (p=0.004), representing a 10.3% reduction. The dose to 100% of hippocampal volume (D_100%_) was 763.33 ± 31.45 cGy for non-coplanar VMAT compared to 899.80 ± 108.56 cGy for TomoTherapy (p=0.004), achieving 15.2% reduction. Both modalities met the RTOG 0933 constraint for D_100%_ (≤900 cGy).

The superior hippocampal sparing achieved by non-coplanar VMAT stems from its ability to deliver dose from superior-inferior beam trajectories through multiple couch angles (0°, 315°, 45°, 90°), which better conforms to the longitudinal hippocampal geometry. This geometric advantage allows steeper dose gradients in the axial direction, creating improved separation between the hippocampal avoidance zone and the whole-brain PTV.

#### 3.2.2 Periocular structures

Bilateral optic nerve maximum doses were significantly lower with non-coplanar VMAT: left 2113.70 ± 218.75 cGy versus 2404.50 ± 433.44 cGy (p=0.045), and right 2071.40 ± 253.06 cGy versus 2398.37 ± 481.59 cGy (p=0.043).

Bilateral eye maximum doses showed improvements with non-coplanar VMAT: left 1556.83 ± 138.72 cGy versus 1981.09 ± 92.80 cGy (p=0.004), and right 1547.29 ± 175.95 cGy versus 2116.07 ± 119.94 cGy (p=0.004), representing 21-27% reductions.

Bilateral lens maximum doses were significantly lower with non-coplanar VMAT: left 350.33 ± 69.26 cGy versus 453.08 ± 101.04 cGy (p=0.041), and right 367.97 ± 73.00 cGy versus 461.58 ± 106.43 cGy (p=0.04).

#### 3.2.3 Other organs at risk

Bilateral cochlear maximum doses were significantly lower with non-coplanar VMAT: left 1739.61 ± 364.00 cGy versus 2054.39 ± 514.79 cGy (p=0.004), and right 1728.54 ± 358.04 cGy versus 2051.19 ± 469.51 cGy (p=0.008), representing approximately 15% reductions.

Bilateral parotid gland mean doses were significantly lower with TomoTherapy: left 218.18 ± 148.67 cGy versus 325.21 ± 94.29 cGy (p=0.034), and right 217.67 ± 133.74 cGy versus 314.48 ± 80.89 cGy (p=0.027).

### 3.3 Treatment efficiency evaluation

**Table 4** presents treatment efficiency comparisons. Total monitor units were 747.69 ± 36.80 MU for non-coplanar VMAT versus 6528.29 ± 488.65 MU for TomoTherapy (p=0.004), representing an 88.5% reduction. Treatment delivery time was 287.33 ± 12.24 seconds for non-coplanar VMAT versus 384.11 ± 28.17 seconds for TomoTherapy (p=0.004), representing a 25.2% reduction.

**Table 4:** Treatment efficiency comparison between TomoTherapy and non-coplanar VMAT.

Parameter	TomoTherapy	Non-coplanar VMAT	p-value
Monitor Units (MU)	6528.29 ± 488.65	747.69 ± 36.80	0.004
Treatment Time (sec)	384.11 ± 28.17	287.33 ± 12.24	0.004

MU, monitor units.

## 4 Discussion

This study provides the first direct comparison of TomoTherapy and TrueBeam non-coplanar VMAT for SCLC patients undergoing HA-PCI with 3-mm margins. Non-coplanar VMAT demonstrated superiority in hippocampal protection, PTV_WB coverage, dose conformity, homogeneity, and treatment efficiency (all p ≤ 0.012).

The 3-mm margin represents an important evolution from RTOG 0933’s 5-mm specification, enabled by modern IGRT capabilities (15–17). This margin reduction increases PTV_WB volume while reducing the hippocampal avoidance zone, but demands steeper dose gradients—transitioning from 900 cGy to 2500 cGy over just 3 mm of tissue.

While TomoTherapy plans in our study exceeded the per-protocol hippocampal dose constraint of 16 Gy, with mean D_max_ of 16.38 Gy, they remained within the acceptable deviation threshold of 17 Gy defined by RTOG 0933. This indicates that TomoTherapy can deliver clinically acceptable hippocampal-sparing WBRT even with reduced 3-mm margins, though achieving per-protocol compliance becomes challenging. Recent systematic evidence (27) demonstrated that narrower field widths (1 cm) provide only modest improvement over 2.5 cm under standard 5-mm margin conditions, with hippocampal D_max_ difference of only 0.27 Gy (1.8%). This suggests that parameter optimization alone may not fully overcome the geometric constraints imposed by reduced margins and predominantly axial beam delivery.

The fundamental advantage of non-coplanar VMAT in this scenario extends beyond merely enabling feasible treatment—as both modalities achieved clinically acceptable outcomes—to providing substantial improvement in hippocampal sparing with per-protocol compliance. NC-VMAT achieved mean hippocampal D_max_ of 13.53 Gy, representing 2.85 Gy (17.4%) absolute dose reduction compared to TomoTherapy, with 2.47 Gy margin below the per-protocol constraint. This clinically meaningful dose reduction stems from the geometric capability of non-coplanar beam arrangements to sculpt dose in the superior-inferior direction, better matching the longitudinal orientation of hippocampal structures. The multiple couch angles (0°, 315°, 45°, 90°) enable beam trajectories that complement the hippocampal geometry, transcending the limitations of predominantly axial delivery while maintaining robust target coverage.

The dosimetric superiority stems from geometric differences between techniques. TomoTherapy’s helical delivery employs predominantly transverse beam trajectories, with limitations in forming steep axial dose gradients. The longitudinally-oriented hippocampus requires precise superior-inferior dose sculpting. Non-coplanar VMAT overcomes this through strategic couch angles (0°, 315°, 45°, 90°), diversifying beam trajectories in three-dimensional space. Couch angle of 90° optimizes superior-inferior dose painting, while oblique angles enable tangential hippocampal avoidance—particularly critical with reduced margins requiring millimeter-scale precision (25).

Non-coplanar VMAT also demonstrated superior PTV_WB coverage with higher V_98%_, V_95%_, and D_98%_ (all p=0.004), better Paddick conformity (0.91 *vs* 0.84, p=0.012), and improved homogeneity (0.20 *vs* 0.27, p=0.004). For organs at risk, non-coplanar VMAT achieved 21-27% lower eye doses (p=0.004) and approximately 15% lower cochlear doses (p ≤ 0.008). TomoTherapy’s parotid sparing advantage (approximately 33% lower, p ≤ 0.034) reflects helical delivery characteristics but represents a less critical endpoint for PCI. Unlike head and neck cancer radiotherapy, where the parotid glands are inevitably irradiated due to their proximity to target volumes and salivary function preservation is therefore a primary clinical concern, PCI targets the whole brain at a relatively low prescription dose (25 Gy in 10 fractions), resulting in parotid doses far below xerostomia thresholds regardless of technique. The parotid doses observed in both techniques (TomoTherapy: 217–218 cGy, non-coplanar VMAT: 314–325 cGy) remain well below the QUANTEC-recommended tolerance thresholds for xerostomia prevention (mean dose<20 Gy for at least one gland, or<25 Gy for both glands) (28). Therefore, while TomoTherapy demonstrated superior parotid sparing, this dosimetric difference is unlikely to translate into meaningful differences in salivary function or quality of life in the PCI setting. The trade-off of modestly higher parotid doses with non-coplanar VMAT is therefore considered clinically acceptable given its substantially superior hippocampal sparing and target coverage.

Treatment efficiency strongly favored non-coplanar VMAT with 88.5% lower monitor units and 25.2% shorter delivery time (both p=0.004). Lower monitor units reduce secondary cancer risk, while shorter treatment reduces intrafraction motion and improves patient comfort.

The clinical implementation of non-coplanar VMAT for HA-PCI requires comprehensive quality assurance protocols. At our institution, HyperArc-based non-coplanar delivery undergoes rigorous verification including collision avoidance protocols for non-coplanar angles, daily cone-beam CT image guidance for all patients receiving advanced radiotherapy techniques, and systematic mechanical accuracy verification. Quality assurance procedures include CBCT isocenter verification, Winston-Lutz tests for gantry, collimator, and couch rotation center alignment, and routine dosimetric QA incorporating full rotational movements of gantry, collimator, and couch. These quality assurance measures ensure safe and accurate delivery of non-coplanar treatments, which is essential given the tight 3-mm hippocampal margins employed in this study.

This study has several limitations. First, the sample size of 10 patients, while comparable to previous dosimetric comparison studies in hippocampal-avoidance radiotherapy (21, 26), limits generalizability. However, the paired study design where each patient serves as their own control enhances statistical power by eliminating inter-patient anatomical variability, and the strong statistical significance (p ≤ 0.012) across multiple endpoints supports the robustness of our findings.

Second, as a planning comparison study, our results represent calculated dose distributions rather than delivered doses. While daily cone-beam CT image guidance at our institution minimizes systematic setup errors, potential discrepancies between planned and delivered doses due to intrafraction motion or positioning uncertainties cannot be entirely excluded.

Third, the TomoTherapy plans represent our institutional clinical planning practice using parameters established through clinical optimization during initial implementation of hippocampal-avoidance WBRT. Treatment planning is inherently dependent on planner expertise, institutional protocols, and optimization strategies, which may vary across institutions. Alternative planning approaches, including different field width configurations or optimization parameters, might yield different dosimetric results. Notably, institution-specific parameters including TomoTherapy modulation factor ranges (1.8-2.4 in this study) and HyperArc template configurations may influence dosimetric outcomes. Furthermore, the HyperArc template used in this study is specific to Varian TrueBeam systems, and institutions with different linear accelerator platforms may require alternative non-coplanar configurations to achieve similar geometric advantages. Our TomoTherapy results, while exceeding per-protocol constraints by 0.38 Gy (2.4%), remained within acceptable deviation thresholds, suggesting that optimization of planning parameters could potentially achieve per-protocol compliance in some cases. However, the fundamental geometric challenge of hippocampal sparing with reduced 3-mm margins using predominantly axial beam delivery persists, and the geometric advantage of non-coplanar beam arrangements for this scenario likely remains across planning variations. Multi-institutional validation with diverse planning systems would further strengthen the generalizability of these findings.

Fourth, the clinical implementation of non-coplanar VMAT presents practical challenges compared to conventional coplanar delivery. Multiple couch rotations increase treatment complexity, delivery time, and quality assurance requirements, while introducing potential collision risks. These workflow considerations may limit routine applicability in high-throughput clinical settings. However, for selected patients where cognitive preservation is particularly critical—such as those with favorable prognosis or younger age—the substantial dosimetric advantages (17.4% hippocampal dose reduction) may justify the additional complexity. Future studies should evaluate clinical feasibility and cost-effectiveness of non-coplanar hippocampal-sparing WBRT in routine practice.

Future prospective studies should evaluate neurocognitive outcomes in patients treated with non-coplanar VMAT versus TomoTherapy to validate the clinical benefit of superior hippocampal sparing demonstrated in this dosimetric comparison.

For institutions with linear accelerator-based systems capable of non-coplanar delivery with appropriate quality assurance programs, this approach represents a promising technique that warrants further clinical investigation for HA-PCI in SCLC patients.

## 5 Conclusion

This study demonstrates that non-coplanar VMAT is superior to TomoTherapy for HA-PCI in SCLC patients using 3-mm margins. Non-coplanar VMAT met RTOG 0933 hippocampal constraints while TomoTherapy exceeded the per-protocol constraint but remained within the acceptable deviation threshold, and simultaneously achieved superior target coverage, conformity, homogeneity, and treatment efficiency. The geometric flexibility of non-coplanar beam arrangements provides superior three-dimensional dose sculpting with reduced margins requiring steep dose gradients.

While these dosimetric advantages are compelling, prospective clinical trials with longitudinal neurocognitive assessment are needed to confirm that superior hippocampal sparing translates to cognitive preservation. For institutions with appropriate linear accelerator capabilities and quality assurance programs, non-coplanar VMAT represents a promising approach for high-precision HA-PCI that warrants clinical validation.

## Data Availability

The original contributions presented in the study are included in the article/Supplementary Material. Further inquiries can be directed to the corresponding author.
